# Successful IVIG Treatment in Neonatal Hemochromatosis Without Extrahepatic Siderosis: A Case Report

**DOI:** 10.3390/reports8020053

**Published:** 2025-04-23

**Authors:** Gwan Yong Lim, Ashta Thashin, Gayathri Joy, Ithamar Cheyne, Małgorzata Mikaszewska-Sokolewicz

**Affiliations:** 1 Anesthesiology and Intensive Care Scientific Circle, Medical University of Warsaw, 02-091 Warsaw, Poland; 2 Clinic of Anesthesiology and Intensive Care, The Children’s Memorial Health Institute, 04-730 Warsaw, Poland

**Keywords:** neonatal hemochromatosis, extrahepatic siderosis, respiratory failure, gestational alloimmune liver disease, intravenous immunoglobulin

## Abstract

**Background and Clinical Significance**: Neonatal hemochromatosis is a rare iron overload disorder that causes severe liver injury in newborns, typically with extrahepatic siderosis. Diagnosis of neonatal hemochromatosis is usually confirmed through a biopsy and MRI, demonstrating deposition of iron and liver failure. However, in severe patients who are not able to undergo biopsy, the diagnostic and management method remains unknown. **Case Presentation**: We present an unusual case of neonatal hemochromatosis without extrahepatic iron deposition in a 9-day-old male who showed signs of liver failure and respiratory distress. This case suggests that when the risks of biopsy outweigh its benefits, a diagnosis may be reached based on clinical evaluation and MRI findings. Early high-dose intravenous immunoglobulin therapy improved liver function and led to recovery, highlighting the need for early therapeutic intervention in neonatal hemochromatosis. **Conclusions**: This case highlights that the absence of extrahepatic siderosis cannot exclude a diagnosis of neonatal hemochromatosis, and high doses of IVIG should be administered promptly when neonatal hemochromatosis is suspected to maximize therapeutic effectiveness.

## 1. Introduction and Clinical Significance

Neonatal hemochromatosis (NH) is a rare and often fatal iron overload disorder during the neonatal period [1]. It serves as the leading cause of cirrhosis and liver failure in this age group. Traditionally, NH has been characterized by severe hepatic injury, with iron accumulating predominantly in the liver and other tissues, while the reticuloendothelial system is unaffected, a pattern that distinguishes it from hereditary hemochromatosis [1,2]. The primary etiology of NH is now thought to be gestational alloimmune liver disease (GALD), in which maternal antibodies induce fetal liver injury, leading to subsequent iron deposition; extrahepatic sclerodermal deposits are often considered to be a process as a secondary consequence [1]. NH is predominantly diagnosed by conducting histopathological and radiological tests. It is differentiated from hereditary hemochromatosis with the aid of a liver biopsy that shows iron accumulation in hepatocytes with reduced involvement of Kupffer cells. However, performing a biopsy in a critical patient still remains doubtful. Radiological tests, such as an MRI, are critical in the diagnosis of NH as they illustrate a low signal intensity of the liver on T2-weighted images, suggesting excessive iron deposition.

In this case report, we describe an unusual variant, i.e., neonatal hemochromatosis without extrahepatic iron deposition on MRI, and underscore the need for a deeper understanding of this spectrum of disorders, especially given the difficulty in management and diagnosis when the patient is too ill to undergo biopsy.

## 2. Case Presentation

The patient, a 9-day-old male newborn, was admitted to the hospital for evaluation of liver enlargement and respiratory failure. He presented with metabolic imbalance, fluid retention, and inflammation, which was due to his respiratory failure secondary to acute liver failure. The patient underwent treatment, which consisted of Duo positive airway pressure and, subsequently, mechanical ventilation as his condition deteriorated. He was the third child born to nonconsanguineous parents and was delivered at 36 weeks of gestation, with a birth weight of 3520 g. The mother had hypothyroidism; otherwise, the patient’s family history was unremarkable. Pregnancy complications included a urinary tract infection at 33 weeks of gestation. Nevertheless, no Group B Streptococcus test was performed, and prophylactic antibiotics were not administered. No other maternal screening (such as COVID-19 or autoimmune antibodies (anti-Ro and anti-La)) was performed. This is notable as GBS may be a contributing etiology to various neonatal infections, such as sepsis, pneumonia, and meningitis, which can worsen liver function and other complications in the neonate. The patient’s Apgar scores were 8 and 9 at 1 and 5 min after birth, respectively, and he was immediately transferred to the neonatal department because of dyspnea.

In the emergency room, the patient exhibited jaundice, facial edema, and multiple bruises, along with anemia, fluid accumulation in the retroperitoneal space, and signs of decreased immunity. The initial vital signs were as follows: blood pressure, 65/35 mmHg; heart rate, 160 beats/min; and respiratory rate, 70/min. An initial laboratory evaluation revealed elevated liver enzyme levels (alanine transaminase: 1145 U/L (reference range: 5–60 U/L); aspartate transaminase: 7216 U/L (reference range: 20–67 U/L); alpha-fetoprotein: 3051 IU/mL (reference range: <8 IU/mL); ferritin: 2363 ng/mL (reference range: 21.81–274.66 ng/mL); D-dimer: 23,188 μg/L (reference range: <500 μg/L); and iron: 274 μg/dL (reference range: 16–128 μg/dL)). Fibrinogen levels were considerably low at a value of 0.6 g/L (reference range: 1.9–3.6 g/L), which subsequently increased to 1.17 g/L. However, triglycerides were consistently low at 55 mg/dL (reference range: 82–259 mg/dL).

**Figure 1:**
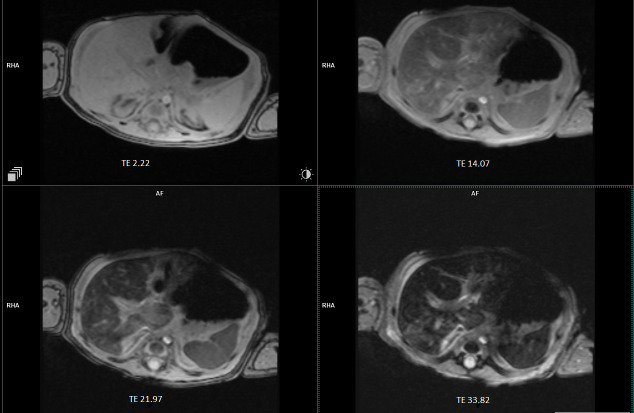
Abdominal MRI showing the decreased signal intensity in the liver parenchyma with no extrahepatic siderosis. MRI: magnetic resonance imaging.

Hemophagocytic lymphohistiocytosis (HLH), gestational alloimmune liver disease (GALD), congenital metabolic disorders, and viral infections, such as CMV, EBV, HSV, and HHV-6, are some of the differential diagnoses that were taken into consideration. The patient was serologically negative for cytomegalovirus, Epstein–Barr virus, herpes simplex virus-1 and -2, human betaherpesvirus 7, varicella zoster virus, adenovirus, enterovirus, parechovirus, and parvovirus B19. The findings were inconclusive for human herpesvirus 6, while positive fungal infection biomarkers were noted; however, the lack of clinical fungal infection signs, sterile cultures, and clinical improvement without antifungals suggested it was most likely a false positive due to contamination. Abdominal magnetic resonance imaging (MRI) revealed decreased signal intensity in the liver parenchyma, with T2-weighted imaging showing no pancreatic abnormalities, suggesting only hepatic siderosis (Figure 1). The thyroid gland MRI was normal, while the brain MRI revealed several 5 mm ring-shaped calcified lesions. Screening for congenital metabolic disorders, such as plasma amino acids, gas chromatography–mass spectrometry (GC-MS), acylcarnitine profile, transferrin isoforms, creatine kinase, and ammonia, was unremarkable, and infectious diseases revealed nondiagnostic findings, ruling out these conditions as primary etiologies of acute liver failure. Furthermore, lactate levels that showed normal values and lack of hallmark radiological findings led to the conclusion that it was unlikely to be of metabolic etiology. The positive response of our patient to receiving IVIG further supported the likelihood of an immunological cause of the disease, resulting in GALD being the most probable diagnosis. Tyrosinemia Type 1 was excluded based on negative succinylacetone test results. Anti-fetal hepatocyte IgG testing was not performed in this case. The collective decision not to conduct liver, salivary gland, and lip biopsies was made by the team of doctors as the patient was in a critical state characterized by severe coagulopathy (prolonged PT and aPTT, markedly reduced fibrinogen, and elevated D-dimer), severe anemia, hemodynamic instability, and respiratory failure. The risks of performing the biopsies, including severe bleeding, anesthesia-related respiratory depression, hypotension, and potential exacerbation of the patient’s respiratory failure, clearly outweighed the potential diagnostic benefits.

Based on the above tests, the patient was finally diagnosed with neonatal hemochromatosis (NH). Due to the extremely high ferritin, intravenous immunoglobulin (IVIG) (1 g/kg) was administered, resulting in a favorable response. Given the good response to IVIG and the patient’s critical state, the medical team decided to avoid invasive procedures, such as exchange transfusion.

Following IVIG administration, the patient’s immunoglobulin G (IgG) levels increased, indicating the therapeutic efficacy of the treatment. The patient’s serological results before and after treatment are summarized in Table 1. Owing to the favorable response to IVIG, this treatment was continuously administered throughout hospitalization in our clinic, until the patient was ultimately discharged after 66 days. Prolonged IVIG was administered due to the risk of household infection exposure. After discharge, the child remained under regular assessment in the immunology clinic. At 6 months, a follow-up assessment was conducted, indicating normal liver function tests and growth parameters, which suggested a positive outcome from the IVIG treatment.

## 3. Discussion

NH is a rare iron overload disease first described in 1957, with a prevalence of <1 per 100,000 births. This disease can be congenital or familial; however, its exact inheritance patterns remain unidentified [1]. The recurrence rate of NH is particularly high in the offspring of women who have previously had an affected child with NH, despite the disease being hetero-paternal. Without early intervention and prophylaxis, the likelihood of children being affected by NH can reach up to 90% [2]. As extrahepatic siderosis was not present, diagnosing the patient was a challenge. However, the patient’s clinical presentation, along with the positive response to the IVIG treatment, supported the diagnosis of NH.

Symptoms of NH typically manifest within 48 h of birth, while intrauterine oligohydramnios, growth restriction, and premature labor are common prenatal findings [1]. NH is the most common etiology of acute liver failure in the neonatal period; however, it is also observed in patients with infections and metabolic disorders, necessitating a thorough differential examination. The diagnosis of HLH was excluded due to normal sCD25 levels and perforin expression. The clinical manifestations of NH are predominantly nonspecific, making the diagnosis challenging. Iron deposition occurs most frequently in the pancreatic parenchyma, followed by the kidneys, thymus, myocardium, trachea, and minor salivary glands [3]. In most cases of NH, iron deposition typically presents in more than two organs, such as both the pancreas and the kidneys.

Gestational alloimmune liver disease (GALD) is a well-known etiology of NH. During pregnancy, maternal IgG crosses the placenta from the twelfth week of gestation, providing the fetus passive humoral immune response. However, if maternal B lymphocytes recognize the fetal antigen as foreign, specific antibodies are produced. In GALD, fetal antigens cross the placenta due to exocytic vesicles or soluble proteins released following apoptosis during rapid hepatic growth [1]. Trans-placental transfer of specific IgG antibodies bound to fetal hepatic antigens triggers a membrane attack complex, leading to direct hepatic damage and secondary iron accumulation in organs, both of which are considered a consequence of the disease, rather than a primary pathological event. Notably, non-GALD diseases account for approximately 2% of all NH cases [1].

Histopathological findings in GALD-induced NH include extensive hepatocyte injuries, while the remaining functioning hepatocytes manifest granular siderosis and giant cell transformation. However, there may be some cases in which no functioning hepatocytes remain. Panlobular parenchymal fibrosis and regenerative nodules are frequently observed, while the portal system is usually unaffected. Parenchymal inflammatory infiltrates, primarily consisting of macrophages and innate immune cells, are abundant [1].

Diagnosis of NH relies on assessing iron levels, abdominal MRI, and biopsies. Abdominal MRI is a key diagnostic tool for NH, as it detects excessive accumulation of iron in the hepatic and extrahepatic tissues. However, some case reports [4,5] have suggested that NH diagnosis is possible even without demonstrating extrahepatic siderosis. Our patient may have experienced a milder form of NH, demonstrating a phenotype of the disease that presented with the absence of extrahepatic siderosis, which differs from the classic presentation of NH. Nevertheless, this feature supports the diagnosis of NH, even in cases where other diagnostic findings are inadequate. Liver biopsy with immunostaining for anti-human C5b-9 complex can also help to diagnose GALD-related NH, but tissue autolysis in stillborn fetuses may reduce the efficacy and accuracy of C5b-9 testing in these cases. Despite this, C5b-9 testing enhances specificity and prevents misdiagnosis, enabling early diagnosis and precise treatment [6]. However, MRI is the preferred primary diagnostic tool as it is less invasive [6,7]. Nonetheless, MRI has certain limitations, including potential false negatives in early or mild siderosis, which may lead to delays in diagnosis.

Early IVIG therapy significantly improves outcomes in neonatal liver failure. A high dose of IVIG is beneficial for treating IgG-mediated diseases, including NH, as it neutralizes immunoglobulins and prevents complement activation. A single dose of IVIG is recommended to be administered to neonates with suspected GALD-induced NH^1^. Side effects of high-dose IVIG are uncommon; however, at a regular dose of 1 g/kg IVIG, an increase in IgG concentration (1400 mg/dL) has been observed [8]. Additional treatments include exchange transfusions, involving the infusion of packed red blood cells in combination with fresh frozen plasma, which have also been used to reduce hyperbilirubinemia [8]. Additionally, antioxidants containing iron-chelating agents and therapeutic cocktails formulated with N-acetylcysteine, vitamin E, selenium, desferrioxamine, and prostaglandin E1 have been used as therapeutic interventions. Liver transplantation is a last resort treatment, but only after nonsurgical therapeutic interventions have been conducted. IVIG has been shown to decrease the need for liver transplantation, as it can improve the prognosis of NH. Collectively, the prognosis of NH worsens without IVIG administration, requiring liver transplantation as further treatment [3].

## 4. Conclusions

Traditionally, extrahepatic siderosis has been considered a key diagnostic criterion for NH. However, this report highlights a case of NH without any evidence of extrahepatic siderosis on MRI, suggesting that the absence of this feature should not exclude the diagnosis of NH. Furthermore, this case report shows that a favorable response to high-dose IVIG treatment during the early course of the disease significantly improves neonatal prognosis. This case reinforces the need for an early diagnosis and prompt treatment of NH regardless of the presence of extrahepatic siderosis. This supports the necessity of IVIG therapy in optimizing the patient’s condition.

## Figures and Tables

**Table 1:** Changes in the patient’s laboratory results during the course of treatment.

	Reference Range	Before Treatment	1–4 Days After Treatment	20 Days After Treatment
**AFP (IU/mL)**	<8	3051	9314.2	22,687
**ALT (U/L)**	5–60	1145	951	9
**AST (U/L)**	20–67	7216	6209	36
**Ferritin (ng/mL)**	21.81–274.66	2363	21,340	1871
**IgG (g/L)**	3.36–10.5	7.5	7.59	16.5
**Direct bilirubin (mg/dL)**	<0.3	1.25	1.13	2.54
**Total bilirubin (mg/dL)**	<1	5.66	3.92	6.91

AFP: alpha fetoprotein, ALT: alanine aminotransferase, AST: aspartate aminotransferase, IgG: immunoglobulin G.

## Data Availability

The data used in this study may be provided by the authors upon reasonable request due to privacy concerns.

## Abbreviations

The following abbreviations are used in this manuscript:

NH	neonatal hemochromatosis
GALD	gestational alloimmune liver disease
MRI	magnetic resonance imaging
IVIG	intravenous immunoglobulin
